# Aqua­(1,10-phenanthroline-κ^2^ *N*,*N*′)(valinato-κ^2^ *N*,*O*)copper(II) nitrate dihydrate

**DOI:** 10.1107/S1600536811047398

**Published:** 2011-11-23

**Authors:** Araceli Tovar-Tovar, Juan-Carlos García-Ramos, Marcos Flores-Alamo, Lena Ruiz-Azuara

**Affiliations:** a Facultad de Química, Universidad Nacional Autónoma de México, Coyoacán 04510, DF, Mexico

## Abstract

In the title compound, [Cu(C_5_H_10_NO_2_)(C_12_H_8_N_2_)(H_2_O)]NO_3_·2H_2_O, the Cu^II^ atom displays a distorted square-pyramidal coordination (τ = 0.03) where the water mol­ecule occupies the apical position and the base is defined by the N atom, one of the O atoms from the valinate ligand, and both phenanthroline N atoms. The phenanthroline chelate ring plane is slightly distorted from planarity (r.m.s. deviation = 0.0057 Å), whereas the five-membered ring formed by the valinate ligand presents an envelope conformation with the N atom being the flap atom. The crystal packing is stabilized by O—H⋯O and N—H⋯O hydrogen-bonding inter­actions, creating a three-dimensional network superstructure.

## Related literature

For investigations related to anti­cancer compounds, see: Ruiz-Azuara (1996 ▶, 1997 ▶). For a description of the geometry of complexes with five-coordinate Cu^II^ atoms, see: Rao *et al.* (1981 ▶); Addison *et al.* (1984 ▶); Le *et al.* (2006 ▶); Dalhus & Görbitz (1999 ▶).
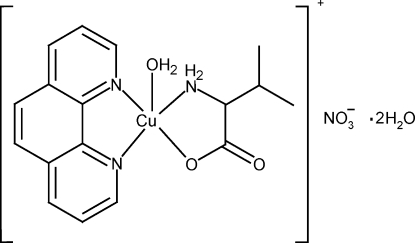


## Experimental

### 

#### Crystal data

[Cu(C_5_H_10_NO_2_)(C_12_H_8_N_2_)(H_2_O)]NO_3_·2H_2_O
*M* *_r_* = 475.94
Triclinic, 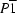

*a* = 7.9020 (19) Å
*b* = 9.610 (3) Å
*c* = 14.327 (4) Å
α = 81.89 (3)°
β = 75.04 (2)°
γ = 87.92 (2)°
*V* = 1040.6 (5) Å^3^
*Z* = 2
Mo *K*α radiation
μ = 1.10 mm^−1^
*T* = 298 K
0.45 × 0.27 × 0.21 mm

#### Data collection

Siemens P4 diffractometer
Absorption correction: ψ scan (*XSCANS*; Siemens, 1993 ▶) *T*_min_ = 0.729, *T*_max_ = 0.794
5563 measured reflections
4551 independent reflections
3807 reflections with *I* > 2σ(*I*)
*R*_int_ = 0.024
3 standard reflections every 97 reflections  intensity decay: 4.9%

#### Refinement

*R*[*F* ^2^ > 2σ(*F* ^2^)] = 0.037
*wR*(*F* ^2^) = 0.094
*S* = 1.06
4551 reflections
297 parameters
18 restraints
H atoms treated by a mixture of independent and constrained refinement
Δρ_max_ = 0.45 e Å^−3^
Δρ_min_ = −0.33 e Å^−3^

### 

Data collection: *XSCANS* (Siemens, 1993 ▶); cell refinement: *XSCANS*; data reduction: *XSCANS*; program(s) used to solve structure: *SIR2004* (Burla *et al.*, 2005 ▶); program(s) used to refine structure: *SHELXL97* (Sheldrick, 2008 ▶); molecular graphics: *ORTEP-3 for Windows* (Farrugia, 1997 ▶); software used to prepare material for publication: *WinGX* (Farrugia, 1999 ▶).

## Supplementary Material

Crystal structure: contains datablock(s) global, I. DOI: 10.1107/S1600536811047398/jj2109sup1.cif

Structure factors: contains datablock(s) I. DOI: 10.1107/S1600536811047398/jj2109Isup2.hkl

Additional supplementary materials:  crystallographic information; 3D view; checkCIF report

## Figures and Tables

**Table 1:** Selected bond lengths (Å)

Cu1—N1	2.0320 (19)
Cu1—N2	1.9975 (19)
Cu1—N3	1.992 (2)
Cu1—O2	1.9349 (16)
Cu1—O3*W*	2.263 (2)

**Table 2:** Hydrogen-bond geometry (Å, °)

*D*—H⋯*A*	*D*—H	H⋯*A*	*D*⋯*A*	*D*—H⋯*A*
O3*W*—H3*A*⋯O1*W*	0.83 (2)	2.02 (2)	2.778 (3)	153 (3)
N3—H01⋯O1*W*	0.87 (3)	2.15 (3)	2.967 (4)	156 (2)
O3*W*—H3*B*⋯O2*W*	0.84 (2)	1.92 (2)	2.753 (3)	171 (3)
O1*W*—H1*A*⋯O4^i^	0.80 (2)	2.02 (2)	2.810 (4)	170 (3)
O1*W*—H1*B*⋯O4^ii^	0.82 (2)	2.19 (2)	2.881 (4)	143 (2)
O1*W*—H1*B*⋯O5^ii^	0.82 (2)	2.48 (2)	3.245 (5)	156 (3)
O2*W*—H2*A*⋯O1^iii^	0.80 (2)	2.05 (2)	2.836 (3)	167 (3)
O2*W*—H2*B*⋯O1^iv^	0.84 (2)	2.02 (2)	2.851 (3)	174 (3)
N3—H02⋯O3^v^	0.82 (3)	2.46 (3)	3.229 (4)	158 (2)
N3—H02⋯O5^v^	0.82 (3)	2.58 (3)	3.168 (4)	130 (2)

Symmetry codes: (i) 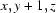; (ii) 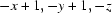; (iii) 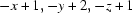; (iv) 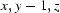; (v) 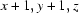.

**Fig. 1.**
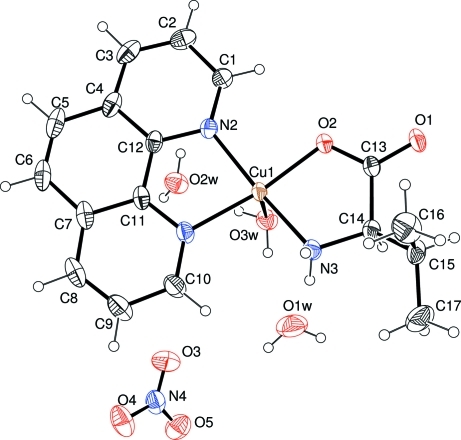
The structure of the title compound, showing the atomic numbering scheme. Non-H atoms are shown with 30% probability displacement ellipsoids.

## supplementary crystallographic information

### Comment

Investigations related to anticancer compounds (Ruiz-Azuara, 1996; 1997) that involve essential metals has been of considerable interest during the last three decades. In this context, we have prepared and crystallized the complex [Cu(H_2_O)(val)(phen)]NO_3_. 2H_2_O (val is valinate and phen is 1,10-phenantroline), (I).

The asymmetric unit consists of one [Cu(H_2_O)(val)(phen)] cationic complex, one nitrate anion and two water molecules. The metallic centre display a distorted square-pyramidal coordination (τ =0.03) where the water molecule occupies the apical position. The base is defined by the N and one of the O atoms from the valinate ligand, and both phenanthroline N atoms. The phenanthroline chelate-ring plane is slightly distorted from planarity (r.m.s. = 0.0057), whereas the five-membered ring formed by the valine ligand (defined by atoms N3, C14, C13, O2 and Cu), presents an envelope conformation on N3 (q2 = 0.2121 and φ = 141.74 °) (Rao *et al.*, 1981). The Cu^II^ ion coordinates two nitrogen atoms of phen and the amino nitrogen and one carboxylate oxygen atoms of L-Val [Cu1–N1 = 2.0320 (19), Cu1–N2 = 1.9975 (19), Cu1–N3 = 1.992 (2), and Cu1–O2 = 1.9349 (16) Å], while one water oxygen atom is axial [Cu1–O3w = 2.263 (2) Å]. The resulting coordination geometries are in a distorted square-pyramidal orientation (figure 1), where N1,N2, N3, O2 and Cu1 for the complex deviate by -0.0079, 0.0085, -0.0085, 0.0079 and 0.2064 Å, respectively, from the least-squares plane (0.84710x+0.53076y+0.02687z = 12.60140) defined by the four ligating atoms N1,N2, N3, and O2.  This indicates that the five atoms in the equatorial positions are approximately coplanar with τ of 0.03 (Addison, *et al.*, 1984). The bond angles observed around the central Cu atom range from 82.13 (8) – 99.01 (8)° in equatorial positions and from 93.90 (8) – 98.54 (8)° for apical positions, showing the angle variability in the geometry adopted by the five coordinate Cu^II^ complexe (Le *et al.*, 2006). The carboxyl group of the amino acid coordinates to Cu^II^*via* one oxygen atom as an unidentate group. Electron delocalization has been observed in the carboxyl group.  However, the bond distances (1.270 (3) Å) between the coordinated oxygen atoms and the carbon atoms are slightly longer (1.254 Å) than those between the uncoordinated oxygen atoms and the carbon atoms as expected (Dalhus & Görbitz, 1999).

The nitrate ion and two water molecules are not involved in the coordination sphere of the Cu ion, but are in the crystal lattice. In the supramolecular network there are O—H···O and N—H···O hydrogen bond interactions and weak O—H···O and N—H···O intermolecular interactions (table 1) that help stabilize crystal packing. The O1w donor-acceptor atom of water molecule solvate interacts with O4 acceptor atom of the nitrate group and the N3 donor atom of the amino group, forming *R*_4_^2^(6) and 2*R*_2_^1^(6) motifs, respectively.  In addition the hydrogen bond formed from the O3w donor atom of the water coordinated to the metal and O2w donor-acceptor atom of the water solvate and O1 acceptor atom of carboxylate group form a C_2_^2^(5) motif. All these interactions lead to infinite three-dimensional network superstructure with base vectors: #1 = [0 1 0], #2 = [1 0 0], #3 = [0 0 1].

### Experimental

1 mmol (0.232 g) of hemi-pentahydrated Cu(NO_3_)_2_ was dissolved in 5 ml of water and mixed with the corresponding amount of 1,10-phenanthroline (1 mmol, 0.180 g) previously dissolved in alcohol (5 ml). To the resulting a deprotonated solution (10 ml) of L-valine (1 mmol, 0.117 g) was added under constant stirring to get a deep-blue product. Further purification was done washing the solid several times with water. The solid was isolated with 95% yield. Single crystals suitable for X-ray diffraction were obtained by slow evaporation of MeOH. Anal. calcd. for C_17_H_24_N_4_O_8_Cu (475.94 g/mol): C, 42.90; H, 5.08; N, 11.77.  Found: C, 42.53; H, 5.11; N, 11.81.

### Refinement

H atoms bonded to N and O atoms were located in difference maps and were refined with free coordinates and *U*_iso_(H) = 1.2Ueq(N) and 1.2Ueq(N). H atoms attached to C atoms were placed in geometrically idealized positions, and refined as riding on their parent atoms, with C—H distances fixed to 0.930 (aromatic CH), 0.960 (methyl CH_3_) and 0.980 Å (methine CH_2_) with *U*_iso_(H) = 1.5 U_eq_(methyl C) or 1.2 U_eq_(C).

### Crystal data

**Table:**

[Cu(C_5_H_10_NO_2_)(C_12_H_8_N_2_)(H_2_O)]NO_3_·2H_2_O	*Z* = 2
*M**_r_* = 475.94	*F*(000) = 494
Triclinic, *P*1	*D*_x_ = 1.519 Mg m^−^^3^
*a* = 7.9020 (19) Å	Mo *K*α radiation, λ = 0.71073 Å
*b* = 9.610 (3) Å	Cell parameters from 43 reflections
*c* = 14.327 (4) Å	θ = 3.4–12.5°
α = 81.89 (3)°	µ = 1.10 mm^−^^1^
β = 75.04 (2)°	*T* = 298 K
γ = 87.92 (2)°	Prism, blue
*V* = 1040.6 (5) Å^3^	0.45 × 0.27 × 0.21 mm

### Data collection

**Table:**

Siemens P4 diffractometer	*R*_int_ = 0.024
graphite	θ_max_ = 27.0°, θ_min_ = 2.1°
2θ/ω scans	*h* = −1→10
Absorption correction: ψ scan (*XSCANS*; Siemens, 1993)	*k* = −12→12
*T*_min_ = 0.729, *T*_max_ = 0.794	*l* = −18→18
5563 measured reflections	3 standard reflections every 97 reflections
4551 independent reflections	intensity decay: 4.9%
3807 reflections with *I* > 2σ(*I*)	

### Refinement

**Table:**

Refinement on *F*^2^	Primary atom site location: structure-invariant direct methods
Least-squares matrix: full	Secondary atom site location: difference Fourier map
*R*[*F*^2^ > 2σ(*F*^2^)] = 0.037	Hydrogen site location: inferred from neighbouring sites
*wR*(*F*^2^) = 0.094	H atoms treated by a mixture of independent and constrained refinement
*S* = 1.06	*w* = 1/[σ^2^(*F*_o_^2^) + (0.0399*P*)^2^ + 0.2653*P*] where *P* = (*F*_o_^2^ + 2*F*_c_^2^)/3
4551 reflections	(Δ/σ)_max_ = 0.002
297 parameters	Δρ_max_ = 0.45 e Å^−^^3^
18 restraints	Δρ_min_ = −0.33 e Å^−^^3^

### Special details

**Table:**

Experimental. IR (KBr disc, cm^-1^): 3426 w, 3285 m, 1623s, 1609 s, 1526 m, 1427 m, 1384 s, 1052 br, 871 m, 725 m.
Geometry. All s.u.'s (except the s.u. in the dihedral angle between two l.s. planes) are estimated using the full covariance matrix. The cell s.u.'s are taken into account individually in the estimation of s.u.'s in distances, angles and torsion angles; correlations between s.u.'s in cell parameters are only used when they are defined by crystal symmetry. An approximate (isotropic) treatment of cell s.u.'s is used for estimating s.u.'s involving l.s. planes.
Refinement. Refinement of *F*^2^ against ALL reflections. The weighted *R*-factor *wR* and goodness of fit *S* are based on *F*^2^, conventional *R*-factors *R* are based on *F*, with *F* set to zero for negative *F*^2^. The threshold expression of *F*^2^ > 2σ(*F*^2^) is used only for calculating *R*-factors(gt) *etc*. and is not relevant to the choice of reflections for refinement. *R*-factors based on *F*^2^ are statistically about twice as large as those based on *F*, and *R*- factors based on ALL data will be even larger.

### Fractional atomic coordinates and isotropic or equivalent isotropic displacement parameters (Å^2^)

**Table:**

	*x*	*y*	*z*	*U*_iso_*/*U*_eq_	
C1	0.7560 (3)	0.9436 (3)	0.53537 (17)	0.0414 (5)	
H1	0.6717	1.0131	0.5489	0.05*	
C2	0.7973 (4)	0.8559 (3)	0.6127 (2)	0.0516 (6)	
H2	0.7417	0.8675	0.6768	0.062*	
C3	0.9208 (4)	0.7526 (3)	0.5934 (2)	0.0532 (7)	
H3	0.9488	0.6936	0.6446	0.064*	
C4	1.0053 (3)	0.7357 (2)	0.4965 (2)	0.0466 (6)	
C5	1.1376 (4)	0.6333 (3)	0.4671 (3)	0.0613 (8)	
H5	1.1738	0.5721	0.5146	0.074*	
C6	1.2105 (4)	0.6237 (3)	0.3725 (3)	0.0622 (8)	
H6	1.2944	0.5547	0.3561	0.075*	
C7	1.1626 (3)	0.7164 (3)	0.2966 (2)	0.0507 (7)	
C8	1.2337 (4)	0.7153 (3)	0.1953 (3)	0.0636 (8)	
H8	1.3207	0.651	0.1736	0.076*	
C9	1.1755 (4)	0.8078 (3)	0.1297 (2)	0.0651 (8)	
H9	1.222	0.8068	0.0631	0.078*	
C10	1.0460 (4)	0.9041 (3)	0.1627 (2)	0.0534 (7)	
H10	1.0061	0.9662	0.1172	0.064*	
C11	1.0336 (3)	0.8191 (2)	0.32296 (19)	0.0386 (5)	
C12	0.9551 (3)	0.8280 (2)	0.42369 (18)	0.0365 (5)	
C13	0.6115 (3)	1.2912 (2)	0.34579 (17)	0.0358 (5)	
C14	0.6524 (3)	1.2917 (2)	0.23517 (17)	0.0405 (5)	
H14	0.5457	1.2598	0.2218	0.049*	
C15	0.6933 (4)	1.4373 (2)	0.17649 (19)	0.0482 (6)	
H15	0.5934	1.4978	0.2001	0.058*	
C16	0.8549 (5)	1.5035 (3)	0.1919 (3)	0.0712 (9)	
H16A	0.8409	1.505	0.2604	0.107*	
H16B	0.8687	1.5979	0.1585	0.107*	
H16C	0.9567	1.4494	0.1666	0.107*	
C17	0.7096 (6)	1.4343 (3)	0.0687 (2)	0.0808 (11)	
H17A	0.8131	1.3833	0.0415	0.121*	
H17B	0.7173	1.5288	0.0354	0.121*	
H17C	0.6085	1.3891	0.0611	0.121*	
N1	0.9771 (3)	0.91087 (19)	0.25678 (14)	0.0400 (4)	
N2	0.8335 (2)	0.93059 (18)	0.44303 (14)	0.0350 (4)	
N3	0.7883 (3)	1.1840 (2)	0.20673 (15)	0.0401 (4)	
N4	0.2440 (4)	0.2073 (3)	0.0776 (2)	0.0687 (7)	
O1	0.5238 (2)	1.38833 (16)	0.38275 (13)	0.0476 (4)	
O2	0.6628 (2)	1.18555 (16)	0.39525 (11)	0.0420 (4)	
O1W	0.6192 (4)	0.9891 (3)	0.11506 (19)	0.0879 (8)	
O3	0.2044 (4)	0.2401 (4)	0.1587 (2)	0.1056 (10)	
O2W	0.6251 (3)	0.6568 (2)	0.41289 (15)	0.0551 (5)	
O4	0.3953 (4)	0.1763 (3)	0.0356 (2)	0.1061 (10)	
O3W	0.5655 (3)	0.91409 (19)	0.31501 (14)	0.0492 (4)	
O5	0.1312 (4)	0.2054 (3)	0.0322 (2)	0.0906 (8)	
Cu1	0.79360 (4)	1.04401 (3)	0.322595 (19)	0.03449 (10)	
H1A	0.547 (3)	1.035 (3)	0.094 (2)	0.052*	
H1B	0.653 (4)	0.925 (2)	0.082 (2)	0.052*	
H2A	0.587 (4)	0.658 (3)	0.4705 (12)	0.052*	
H2B	0.591 (4)	0.581 (2)	0.4010 (18)	0.052*	
H02	0.885 (4)	1.221 (3)	0.188 (2)	0.041*	
H3A	0.572 (4)	0.908 (3)	0.2571 (12)	0.052*	
H01	0.752 (4)	1.144 (3)	0.165 (2)	0.041*	
H3B	0.578 (4)	0.832 (2)	0.3410 (16)	0.052*	

### Atomic displacement parameters (Å^2^)

**Table:**

	*U*^11^	*U*^22^	*U*^33^	*U*^12^	*U*^13^	*U*^23^
C1	0.0417 (13)	0.0409 (12)	0.0405 (12)	−0.0020 (10)	−0.0096 (10)	−0.0033 (9)
C2	0.0562 (16)	0.0562 (15)	0.0420 (13)	−0.0161 (13)	−0.0152 (12)	0.0040 (11)
C3	0.0598 (17)	0.0465 (14)	0.0583 (16)	−0.0121 (12)	−0.0331 (14)	0.0134 (12)
C4	0.0440 (14)	0.0351 (12)	0.0664 (17)	−0.0038 (10)	−0.0296 (13)	0.0036 (11)
C5	0.0549 (17)	0.0399 (13)	0.099 (3)	0.0068 (12)	−0.0439 (18)	0.0021 (14)
C6	0.0470 (16)	0.0438 (14)	0.103 (3)	0.0183 (12)	−0.0325 (17)	−0.0148 (15)
C7	0.0365 (13)	0.0360 (12)	0.082 (2)	0.0076 (10)	−0.0166 (13)	−0.0149 (12)
C8	0.0462 (16)	0.0553 (16)	0.087 (2)	0.0151 (13)	−0.0041 (15)	−0.0294 (16)
C9	0.0649 (19)	0.0583 (17)	0.0625 (18)	0.0075 (15)	0.0063 (15)	−0.0206 (14)
C10	0.0624 (17)	0.0427 (13)	0.0468 (14)	0.0065 (12)	0.0013 (13)	−0.0088 (11)
C11	0.0315 (11)	0.0279 (10)	0.0568 (14)	0.0023 (9)	−0.0111 (10)	−0.0076 (9)
C12	0.0338 (11)	0.0263 (10)	0.0508 (13)	−0.0007 (8)	−0.0154 (10)	−0.0012 (9)
C13	0.0386 (12)	0.0268 (10)	0.0420 (12)	0.0026 (9)	−0.0095 (10)	−0.0067 (8)
C14	0.0457 (13)	0.0302 (10)	0.0450 (13)	0.0044 (9)	−0.0121 (11)	−0.0031 (9)
C15	0.0595 (16)	0.0289 (11)	0.0527 (14)	0.0057 (11)	−0.0134 (12)	0.0031 (10)
C16	0.081 (2)	0.0494 (16)	0.077 (2)	−0.0162 (16)	−0.0099 (18)	−0.0042 (15)
C17	0.125 (3)	0.0559 (18)	0.0584 (19)	−0.001 (2)	−0.031 (2)	0.0153 (14)
N1	0.0427 (11)	0.0303 (9)	0.0430 (11)	0.0062 (8)	−0.0047 (9)	−0.0045 (8)
N2	0.0361 (10)	0.0297 (8)	0.0388 (10)	0.0011 (7)	−0.0098 (8)	−0.0025 (7)
N3	0.0490 (12)	0.0303 (9)	0.0372 (10)	0.0061 (9)	−0.0058 (9)	−0.0037 (8)
N4	0.0638 (17)	0.0525 (14)	0.0730 (19)	0.0042 (12)	0.0036 (15)	0.0074 (13)
O1	0.0562 (11)	0.0321 (8)	0.0531 (10)	0.0152 (8)	−0.0100 (8)	−0.0125 (7)
O2	0.0544 (10)	0.0344 (8)	0.0368 (8)	0.0153 (7)	−0.0114 (7)	−0.0076 (6)
O1W	0.116 (2)	0.101 (2)	0.0612 (15)	−0.0010 (17)	−0.0442 (15)	−0.0188 (13)
O3	0.115 (2)	0.133 (3)	0.0578 (15)	0.011 (2)	−0.0038 (15)	−0.0138 (16)
O2W	0.0665 (13)	0.0449 (10)	0.0520 (11)	−0.0081 (9)	−0.0089 (10)	−0.0100 (9)
O4	0.0671 (17)	0.103 (2)	0.131 (3)	0.0137 (15)	0.0094 (17)	−0.0271 (19)
O3W	0.0577 (11)	0.0433 (9)	0.0466 (10)	−0.0016 (8)	−0.0122 (9)	−0.0078 (8)
O5	0.091 (2)	0.0889 (19)	0.0850 (18)	−0.0104 (15)	−0.0212 (16)	0.0139 (14)
Cu1	0.04126 (17)	0.02603 (13)	0.03374 (15)	0.00937 (10)	−0.00668 (11)	−0.00374 (9)

### Geometric parameters (Å, °)

**Table:**

C1—N2	1.328 (3)	C14—C15	1.526 (3)
C1—C2	1.395 (4)	C14—H14	0.98
C1—H1	0.93	C15—C17	1.520 (4)
C2—C3	1.372 (4)	C15—C16	1.526 (4)
C2—H2	0.93	C15—H15	0.98
C3—C4	1.407 (4)	C16—H16A	0.96
C3—H3	0.93	C16—H16B	0.96
C4—C12	1.399 (3)	C16—H16C	0.96
C4—C5	1.433 (4)	C17—H17A	0.96
C5—C6	1.344 (5)	C17—H17B	0.96
C5—H5	0.93	C17—H17C	0.96
C6—C7	1.426 (4)	Cu1—N1	2.0320 (19)
C6—H6	0.93	Cu1—N2	1.9975 (19)
C7—C11	1.410 (3)	Cu1—N3	1.992 (2)
C7—C8	1.416 (5)	N3—H02	0.82 (3)
C8—C9	1.358 (5)	N3—H01	0.87 (3)
C8—H8	0.93	N4—O3	1.207 (4)
C9—C10	1.390 (4)	N4—O5	1.233 (4)
C9—H9	0.93	N4—O4	1.238 (4)
C10—N1	1.326 (3)	Cu1—O2	1.9349 (16)
C10—H10	0.93	O1W—H1A	0.802 (15)
C11—N1	1.353 (3)	O1W—H1B	0.822 (15)
C11—C12	1.430 (4)	O2W—O2W	0
C12—N2	1.357 (3)	O2W—H2A	0.804 (15)
C13—O1	1.239 (3)	O2W—H2B	0.840 (15)
C13—O2	1.270 (3)	Cu1—O3W	2.263 (2)
C13—C14	1.533 (3)	O3W—H3A	0.828 (15)
C14—N3	1.484 (3)	O3W—H3B	0.841 (15)
			
N2—C1—C2	121.9 (2)	C16—C15—C14	112.7 (2)
N2—C1—H1	119	C17—C15—H15	107.1
C2—C1—H1	119	C16—C15—H15	107.1
C3—C2—C1	119.4 (3)	C14—C15—H15	107.1
C3—C2—H2	120.3	C15—C16—H16A	109.5
C1—C2—H2	120.3	C15—C16—H16B	109.5
C2—C3—C4	120.2 (2)	H16A—C16—H16B	109.5
C2—C3—H3	119.9	C15—C16—H16C	109.5
C4—C3—H3	119.9	H16A—C16—H16C	109.5
C12—C4—C3	116.4 (2)	H16B—C16—H16C	109.5
C12—C4—C5	118.2 (3)	C15—C17—H17A	109.5
C3—C4—C5	125.4 (2)	C15—C17—H17B	109.5
C6—C5—C4	121.4 (3)	H17A—C17—H17B	109.5
C6—C5—H5	119.3	C15—C17—H17C	109.5
C4—C5—H5	119.3	H17A—C17—H17C	109.5
C5—C6—C7	121.7 (2)	H17B—C17—H17C	109.5
C5—C6—H6	119.1	C10—N1—C11	118.6 (2)
C7—C6—H6	119.1	C10—N1—Cu1	129.92 (18)
C11—C7—C8	116.0 (3)	C11—N1—Cu1	111.50 (15)
C11—C7—C6	118.3 (3)	C1—N2—C12	118.8 (2)
C8—C7—C6	125.7 (3)	C1—N2—Cu1	128.22 (16)
C9—C8—C7	120.3 (2)	C12—N2—Cu1	112.97 (15)
C9—C8—H8	119.8	C14—N3—Cu1	109.71 (14)
C7—C8—H8	119.8	C14—N3—H02	109.7 (19)
C8—C9—C10	119.4 (3)	Cu1—N3—H02	106.4 (19)
C8—C9—H9	120.3	C14—N3—H01	103.7 (18)
C10—C9—H9	120.3	Cu1—N3—H01	109.8 (18)
N1—C10—C9	122.6 (3)	H02—N3—H01	117 (3)
N1—C10—H10	118.7	O3—N4—O5	119.8 (3)
C9—C10—H10	118.7	O3—N4—O4	123.2 (4)
N1—C11—C7	123.0 (2)	O5—N4—O4	117.0 (3)
N1—C11—C12	117.17 (19)	C13—O2—Cu1	116.53 (15)
C7—C11—C12	119.8 (2)	H1A—O1W—H1B	109 (2)
N2—C12—C4	123.2 (2)	H2A—O2W—H2B	106 (2)
N2—C12—C11	116.22 (19)	Cu1—O3W—H3A	109 (2)
C4—C12—C11	120.5 (2)	Cu1—O3W—H3B	108 (2)
O1—C13—O2	123.5 (2)	H3A—O3W—H3B	105.7 (19)
O1—C13—C14	119.28 (19)	O2—Cu1—N3	84.00 (8)
O2—C13—C14	117.12 (18)	O2—Cu1—N2	92.38 (7)
N3—C14—C15	114.5 (2)	N3—Cu1—N2	168.37 (9)
N3—C14—C13	108.83 (18)	O2—Cu1—N1	166.85 (8)
C15—C14—C13	113.78 (19)	N3—Cu1—N1	99.01 (8)
N3—C14—H14	106.4	N2—Cu1—N1	82.13 (8)
C15—C14—H14	106.4	O2—Cu1—O3W	98.54 (8)
C13—C14—H14	106.4	N3—Cu1—O3W	95.92 (9)
C17—C15—C16	111.1 (3)	N2—Cu1—O3W	95.55 (8)
C17—C15—C14	111.4 (2)	N1—Cu1—O3W	93.90 (8)
			
N2—C1—C2—C3	−0.3 (4)	C7—C11—N1—Cu1	−179.17 (19)
C1—C2—C3—C4	0.2 (4)	C12—C11—N1—Cu1	1.5 (3)
C2—C3—C4—C12	−0.4 (4)	C2—C1—N2—C12	0.7 (3)
C2—C3—C4—C5	179.2 (3)	C2—C1—N2—Cu1	179.91 (18)
C12—C4—C5—C6	−1.0 (4)	C4—C12—N2—C1	−0.9 (3)
C3—C4—C5—C6	179.4 (3)	C11—C12—N2—C1	179.9 (2)
C4—C5—C6—C7	1.2 (5)	C4—C12—N2—Cu1	179.74 (18)
C5—C6—C7—C11	−0.8 (4)	C11—C12—N2—Cu1	0.5 (2)
C5—C6—C7—C8	179.2 (3)	C15—C14—N3—Cu1	149.15 (18)
C11—C7—C8—C9	−1.1 (4)	C13—C14—N3—Cu1	20.5 (2)
C6—C7—C8—C9	179.0 (3)	O1—C13—O2—Cu1	178.54 (18)
C7—C8—C9—C10	0.3 (5)	C14—C13—O2—Cu1	2.4 (3)
C8—C9—C10—N1	0.8 (5)	C13—O2—Cu1—N3	8.10 (18)
C8—C7—C11—N1	0.9 (4)	C13—O2—Cu1—N2	177.01 (18)
C6—C7—C11—N1	−179.1 (2)	C13—O2—Cu1—N1	112.1 (3)
C8—C7—C11—C12	−179.8 (2)	C13—O2—Cu1—O3W	−87.02 (18)
C6—C7—C11—C12	0.2 (4)	C14—N3—Cu1—O2	−16.23 (17)
C3—C4—C12—N2	0.8 (3)	C14—N3—Cu1—N2	−88.6 (4)
C5—C4—C12—N2	−178.8 (2)	C14—N3—Cu1—N1	176.68 (17)
C3—C4—C12—C11	180.0 (2)	C14—N3—Cu1—O3W	81.77 (17)
C5—C4—C12—C11	0.4 (3)	C1—N2—Cu1—O2	13.0 (2)
N1—C11—C12—N2	−1.4 (3)	C12—N2—Cu1—O2	−167.75 (16)
C7—C11—C12—N2	179.3 (2)	C1—N2—Cu1—N3	84.5 (4)
N1—C11—C12—C4	179.3 (2)	C12—N2—Cu1—N3	−96.2 (4)
C7—C11—C12—C4	0.0 (3)	C1—N2—Cu1—N1	−179.0 (2)
O1—C13—C14—N3	168.0 (2)	C12—N2—Cu1—N1	0.24 (15)
O2—C13—C14—N3	−15.7 (3)	C1—N2—Cu1—O3W	−85.8 (2)
O1—C13—C14—C15	38.9 (3)	C12—N2—Cu1—O3W	93.43 (16)
O2—C13—C14—C15	−144.7 (2)	C10—N1—Cu1—O2	−114.1 (4)
N3—C14—C15—C17	61.3 (3)	C11—N1—Cu1—O2	65.0 (4)
C13—C14—C15—C17	−172.6 (3)	C10—N1—Cu1—N3	−11.8 (3)
N3—C14—C15—C16	−64.4 (3)	C11—N1—Cu1—N3	167.33 (17)
C13—C14—C15—C16	61.7 (3)	C10—N1—Cu1—N2	179.9 (2)
C9—C10—N1—C11	−1.0 (4)	C11—N1—Cu1—N2	−0.96 (16)
C9—C10—N1—Cu1	178.1 (2)	C10—N1—Cu1—O3W	84.8 (2)
C7—C11—N1—C10	0.1 (4)	C11—N1—Cu1—O3W	−96.04 (17)
C12—C11—N1—C10	−179.2 (2)		

### Hydrogen-bond geometry (Å, °)

**Table:**

*D*—H···*A*	*D*—H	H···*A*	*D*···*A*	*D*—H···*A*
O3W—H3A···O1W	0.83 (2)	2.02 (2)	2.778 (3)	153 (3)
N3—H01···O1W	0.87 (3)	2.15 (3)	2.967 (4)	156 (2)
O3W—H3B···O2W	0.84 (2)	1.92 (2)	2.753 (3)	171 (3)
O1W—H1A···O4^i^	0.80 (2)	2.02 (2)	2.810 (4)	170 (3)
O1W—H1B···O4^ii^	0.82 (2)	2.19 (2)	2.881 (4)	143 (2)
O1W—H1B···O5^ii^	0.82 (2)	2.48 (2)	3.245 (5)	156 (3)
O2W—H2A···O1^iii^	0.80 (2)	2.05 (2)	2.836 (3)	167 (3)
O2W—H2B···O1^iv^	0.84 (2)	2.02 (2)	2.851 (3)	174 (3)
N3—H02···O3^v^	0.82 (3)	2.46 (3)	3.229 (4)	158 (2)
N3—H02···O5^v^	0.82 (3)	2.58 (3)	3.168 (4)	130 (2)

Symmetry codes:  (i) *x*, *y*+1, *z*; (ii) −*x*+1, −*y*+1, −*z*; (iii) −*x*+1, −*y*+2, −*z*+1; (iv) *x*, *y*−1, *z*; (v) *x*+1, *y*+1, *z*.
